# Evaluation of Respiratory Particle Emission during Otorhinolaryngological Procedures in the Context of the SARS-CoV-2 Pandemic

**DOI:** 10.3390/diagnostics12071603

**Published:** 2022-06-30

**Authors:** Reinhard Veltrup, Stefan Kniesburges, Michael Döllinger, Sebastian Falk, Sarina K. Mueller

**Affiliations:** Department of Otorhinolaryngology, Head and Neck Surgery, University Hospital Erlangen, Friedrich–Alexander-University Erlangen–Nürnberg, 91054 Erlangen, Germany; stefan.kniesburges@uk-erlangen.de (S.K.); michael.doellinger@uk-erlangen.de (M.D.); sebastian.falk@uk-erlangen.de (S.F.); sarina.mueller@uk-erlangen.de (S.K.M.)

**Keywords:** respiratory particles, laryngoscopy, rhinoscopy, otoscopy, sonography, pharyngoscopy, SARS-CoV-2, face masks, high-speed visualization, optical particle sizer

## Abstract

Understanding the risk of infection by routine medical examination is important for the protection of the medical personnel. In this study we investigated respiratory particles emitted by patients during routine otolaryngologic procedures and assessed the risks for the performing physician. We developed two experimental setups to measure aerosol and droplet emission during rigid/flexible laryngoscopy, rhinoscopy, pharyngoscopy, otoscopy, sonography and patient interview for subjects with and without masks. A high-speed-camera setup was used to detect ballistic droplets (approx. > 100 µm) and an aerosol-particle-sizer was used to detect aerosol particles in the range of 0.3 µm to 10 µm. Aerosol particle counts were highly increased for coughing and slightly increased for heavy breathing in subjects without masks. The highest aerosol particle counts occurred during rigid laryngoscopy. During laryngoscopy and rhinoscopy, the examiner was exposed to increased particle emission due to close proximity to the patient’s face and provoked events such as coughing. However, even during sonography or otoscopy without a mask, aerosol particles were expelled close to the examiner. The physician’s exposure to respiratory particles can be reduced by deliberate choice of examination technique depending on medical indication and the use of appropriate equipment for the examiners and the patients (e.g., FFP2 masks for both).

## 1. Introduction

The SARS-CoV-2 virus pandemic has been a big challenge for the medical sector all around the globe. An effective health care system and especially the protection of the medical staff are crucial elements in combating this pandemic, as this occupational category belongs to the group of people with the highest risk of exposure [1]. In an observational cohort study in the UK and the USA, an increased risk of infection with COVID-19 was found for front-line medical workers (nurses and physicians), especially when availability of personal protective equipment (PPE) was insufficient [2,3]. The risk of infection for a medical worker increases with each contact to infected patients and the closer the contact, the higher the risk. Otorhinolaryngological (ENT) procedures are particularly critical as they often have to be conducted with the examiner’s head very close in front of the patient’s face often with risk to provoke choking, sneezing or coughing exposing the examiner to high viral loads [4]. A comprehensive understanding of the special transmission pathways during ENT procedures is necessary in order to take effective preventive measures. It is known that SARS-CoV-2 virus transmits via large droplets as well as small aerosol particles emitted from the mouth and nose [5]. The size of potentially virus-laden aerosol particles starts at the virus size which was determined between 0.06–0.14 µm [6]. The typical size range of aerosol particles was detected between 0.1–1 µm depending on many factors, such as the site of origin in the body, the specific respiratory activity (RA), and the environmental conditions, so the stated sizes vary in literature [7,8,9]. In contrast, particles with a diameter of more than 5 µm are generally referred to as droplets. Infectious risk for typical respiratory activities has been assessed and modeled by Schijven et al. and Wang et al. which both showed highest expelled respiratory fluid volumes for sneezing, followed by coughing, singing, speaking and breathing [10,11]. Thereby, the droplets follow a ballistic trajectory whereas aerosol particles hover in the air and may accumulate in closed rooms to a critical density [12]. In contrast to breathing, speech, spontaneous coughing and sneezing [5], it is unknown so far how much emission is produced during different ENT procedure by e.g., forcing strong respiratory defense reactions as choking/coughing during laryngoscopy or sneezing during rhinoscopy.

Various methods for analyzing respiratory particles have been often described in literature and reviewed by Mahjoub Mohammed Merghani et al. [13]. Large droplets are detected mainly by high-speed video analysis in high contrast settings, e.g., using laser light sheet [14,15,16] or white light illumination of the airborne droplets in front of black background [17,18,19,20]. These techniques allow the analyzation of the RAs non-intrusively and under realistic conditions but with the disadvantage that it is challenging to quantify absolute values accurately, e.g., from droplet counts. Han et al. [21] and Zayas et al. [22] used Laser refraction systems to measure particles with diameters up to 1 mm directly in-front of subjects mouths. Another approach is to collect dry residues of RAs with filters [23,24]. Xie et al. used a sealed chamber with water sensitive paper strips attached to the inside walls and analyzed the stains forming when subjects coughed or spoke into the chamber [25].

Mostly intrusive methods are used when aerosol particles are to be measured down to the sub-micrometer range [13]. In a study by Workman et al. (2020), an optical particle sizer was used to detect aerosol particles from subjects that were, inter alia, coughing, heavily breathing, sneezing and speaking and calculated the differences between the average particle concentrations of the task and background noise [26]. In 2009, Morawska et al. measured dilution corrected aerosol particle size distribution for similar RAs within a closed looped wind channel in combination with an aerodynamic particle sizer [7]. In another study, Papineni et al. (1997) used an optical particle counter (OPC) inside a HEPA filtered biological safety cabinet to detect aerosol particles produced by five subjects in a diameter range of 0.6–2.5 µm [9].

Regardless of particle size and measuring technique, the majority of studies focus on basic spontaneous RAs. The aim of this study is to investigate the expulsion of respiratory particles (ballistic droplets and aerosol particles) produced during six ENT procedures. Additionally, we tested the practicability and filtering effectiveness of surgical and FFP2 masks worn by the patients performing the ENT procedures. The results show the specific emission characteristic, alternatives and possible improvements for protective equipment during the procedures.

## 2. Materials and Methods

### 2.1. Measurement Techniques

This was an IRB approved study by the local ethics committee of the University of Erlangen-Nürnberg (FAU). In this study a combination of high-speed imaging (large droplet measurements) and an optical particle sizer (OPS, aerosol particle measurements) was used in a clinical setting. This combined setting was chosen as the diameter of potentially infective particles emitted from humans cover at least four orders of magnitude. Since it is impossible to measure the whole bandwidth of particles with one technique, we use the two measurement techniques to detect small aerosol particles in the range between 0.3 µm and 10 µm and large droplets reaching from approx. 100 µm to 2 mm. The gap between 10 µm and 100 µm results from the compromise between the cameras large image section and the spatial resolution. However, this compromise is reasonable, since a large range of particles with aerosol and ballistic properties can still be resolved. All subjects underwent otorhinolaryngological examinations in a clinical setting without discrepancy to routine procedures. Details are described in the following.

### 2.2. Subjects

A total of six subjects from the scientific and medical staff of the University Hospital Erlangen were recruited (five male/one female, aged between 27 and 41, 100% non-smoker, 100% healthy, 100% no drug use). The female was only available for the aerosol particle detection measurements. A recruitment of more and external subjects was not possible due to the entrance restrictions of the hospital. Therefore, the sample size in this study is not estimated. The reason for the male majority is that the amount of expelled respiratory particles was found to be larger for males displaying the worst case scenario [27]. Thus, we focus on descriptive statistics and omit calculating significance values, due to this small number of subjects. All subjects had no active respiratory infections and were SARS-CoV-2 negative. There were no active allergic symptoms in any subject during the time point of measurement.

### 2.3. Large Droplet Measurements

In total, six typical ENT procedures were analyzed with regard to their large droplet generation using high-speed imaging. Five were performed in sedentary and one in supine position in the research lab of the phoniatrics department of the University Hospital Erlangen. The following procedures were performed in up to three constellations: without a mask, with a surgical mouth-nose covering (MNC) (Voit ^®^ Face Mask IIR, REF 301 SN 14683 Type IIR) or an FFP2 mask without valve (KINGFA KF-A F10(SC) FFP2 NR CE 0598, EN 149: 2001 + A1: 2009). These masks were selected because they are widely used in the population and are EU certified. Otrisal et al. has shown that there are differences between the various mask standards used worldwide (FFP2, N95, KN95), however, the filtering efficiencies of certified versions of these products are in the same order of magnitude [28]. Every paradigm was performed once per subject.

1. **Patient interview** in sedentary position with subjects reading aloud the standardized text “The north wind and the sun” (German version), equivalent to Rainbow passage, with and without a mask of both types (surgical and FFP2) for 6.6 s.
2. **Pharyngoscopy** in sedentary position without a mask, where the subject’s pharynx is inspected through the mouth with a mirror.
3. **Laryngoscopy** in sedentary position, performed orally and nasally. Orally with a rigid endoscope and nasally with a flexible endoscope. During the task with the rigid endoscope, the physician held the subject’s tongue with a paper tissue. The task with the flexible endoscope was additionally performed with an MNC.
4. **Rhinoscopy** in sedentary position with a rigid endoscope, once performed typically without a mask and once with the subject wearing an MNC with a small hole through which the endoscope was passed through.
5. **Otoscopy** in sedentary position with MNC. The physician inspects the subject’s ear with an otoscope. Since it is common for patients to speak or cough during otoscopy and sonography, subjects were saying aloud the phrase “Stay healthy” and afterwards coughed once within a measuring cycle to simulate the worst-case scenario. The phrase was chosen for its benchmark status [14,29]. For the whole tasks, two cycles of “Stay healthy” and coughing were executed by each subject.
6. **Sonography** of the throat in supine position wearing no mask, an MNC or an FFP2 mask. Subjects speaking/coughing pattern was analogue to otoscopy. The physician positioned next to the subject’s head.

An overview of the performed droplet experiments is shown in Table 1. Note that not every task was performed in all three ways. Pharyngoscopy and oral laryngoscopy were executed only without mask since there is no practical alternative with a mask. Otoscopy was only performed with masks since there is no clinical indication or limitation regarding the procedure for the patient to wear none.

**Figure 1:**
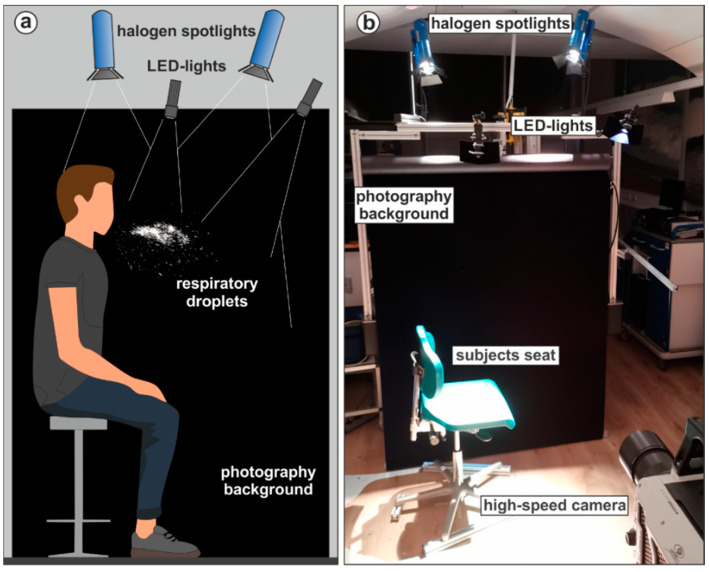
(**a**) Concept figure of the droplet test stand from the high-speed camera perspective. In total, two halogen spotlights and two LED lights illuminated the area in front of the subjects; (**b**) photography of the implemented test stand without subject.

**Figure 2:**
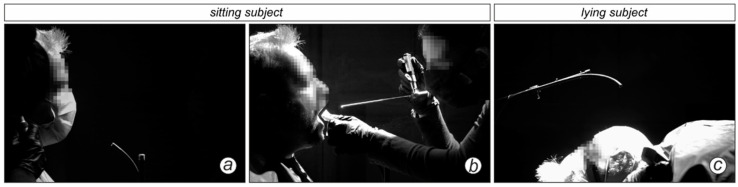
High-speed camera view on three exemplary tasks. (**a**) Otoscopy; (**b**) Rigid laryngoscopy through the mouth without a mask. (**c**) Sonography (supine) with MNC.

**Figure 3:**
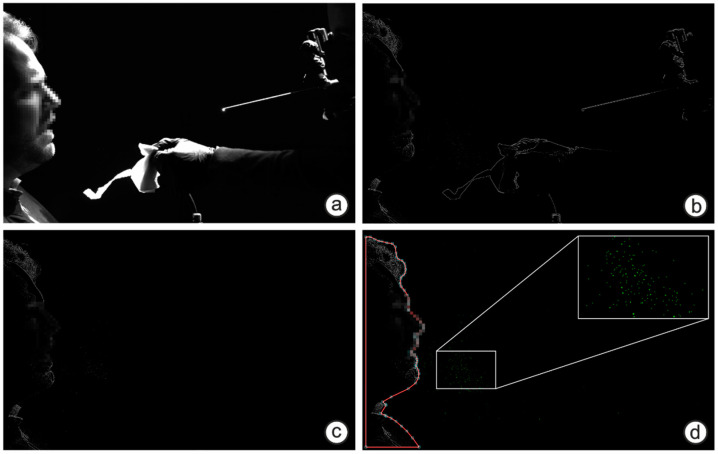
Image processing pipeline for one exemplary frame; (**a**) Unedited image with subject on the left and physician on the right handside; (**b**) Edge high pass filtered image from the software PCC 2.6 (**c**) Image where the physician was cut out with Sensarea (**d**) Masked subject and detected/tracked particles (green). An enlarged image section of the tracked droplets is shown in the upper right corner.

The measuring principle for the droplet experiments was analogue to Mueller et al. (2021) [29] with an optimized stationary setup shown in Figure 1. Subjects were recorded in front of a professional black photography background. The camera was positioned perpendicularly to the subject’s sagittal plane. The framerate of the industrial high-speed camera v2511 (Vision research) was 1000 frames per second (fps) at a resolution of 1280 × 800 pixel and the recording duration per video was 6.6 s. The whole image comprises a region of 60 × 37 cm^2^, which results in spatial resolution of approx. 470 µm per pixel. Smaller droplets can be visualized but not quantified in terms of their diameter [15]. However, this is not a limitation as the focus of this study is on particle counts and not size distributions. Exemplary camera views for two sedentary tasks and one supine task are shown in Figure 2. The subject and the physician were arranged in order to avoid the occlusion of the camera view by the physician during and after the task. The resulting videos were post-processed by an edge high-pass filter in the camera software PCC 2.6 (Vision Research, Version 2.6.749.0, Wayne (New Jersey), USA) to maximize contrast. Afterwards the physician was cut out of the videos with the open-source software Sensarea (Gipsa Lab, Institute of Engineering Univ. Grenoble Alpes, Version 1.12.1, Grenoble, France). The visible droplets in the video were detected and tracked with an in-house particle-tracking software used in Kniesburges et al. (2021) and the resulting data was subsequently analyzed with Mathworks Matlab (Version R2020b). Only droplets that were visible for at least 10 frames were included. The image processing pipeline for one exemplary frame is shown in Figure 3.

### 2.4. Aerosol Particle Measurements

The test stand for large droplets was not viable for measuring the aerosol output during the ENT procedures directly because the physician blocked the area between the funnel of the OPS and the subject. Therefore, we have analyzed which processes occur during the ENT procedures and investigated them on a representative basis. We extracted the following basic RAs from the ENT procedures: heavy breathing, speaking, vocalizing a single tone and coughing (Table 2).

**Figure 4:**
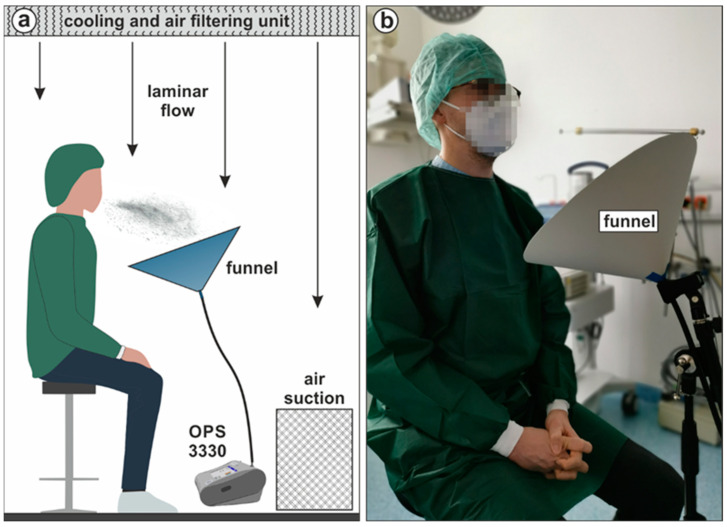
(**a**) Concept drawing of the aerosol test stand in the OR. 2-stage supply air treatment (F7 and F9 filter), temperated air flows laminarly from the ceiling to the floor and is extracted via the air suction unit. Respiratory aerosols expelled by the subject during the procedures are sucked in by the OPS and counted (**b**) Photography of a subject in front of the inlet measuring funnel.

The aerosol experiments were carried out in a clean operating room (OR) in the Department of Otorhinolaryngology of the University Hospital Erlangen, using a TSI Incorporated Optical Particle Sizer Model 3330 (OPS) with a measuring range of 0.3 µm to 10 µm. A concept drawing of the test stand is shown in Figure 4. The air conditioning system has a two-stage supply air treatment (first filter stage F7/second filter stage F9). The air is temperated to 22 °C and flows laminary from the ceiling to the floor at a speed of approx. 0.18 m/s and is extracted via the air suction unit in the wall, near the ground. A nine-hour reference measurement in the empty OR was carried out and showed a mean value of 4.3 aerosol particles/min. The 24 references taken in between the single measuring runs yielded 4.0 aerosol particles/min. This shows that the presence of the staff and subjects in the OR who wore fusel-free and sterile surgical clothes do not create additional pollution that would have increased the background noise. The OPS was connected to a funnel via a 92 cm conductive tube. The funnel has a diameter of 40 cm which tapers down to 1.5 cm and was positioned at an angle of approx. 35° to its vertical axis. Subjects were placed in sedentary position approx. 30 cm in front of the funnel and performed the RAs for one minute, once wearing no mask and once wearing an FFP2 mask. After every task, a one-minute reference measurement was conducted to check background particle count of the OR and to compare the values to the reference. The resulting data was processed within the OPS software Aerosol Instrument Manager Software (TSI Incorporated, Version 9.0.0.0, Shoreview, MN, USA) and Microsoft Excel 2019 MSO 32-bit (Microsoft Corporation, Version 16.0.10382.20010, Redmond, WA, USA).

## 3. Results

### 3.1. Droplet Emission

**Figure 5:**
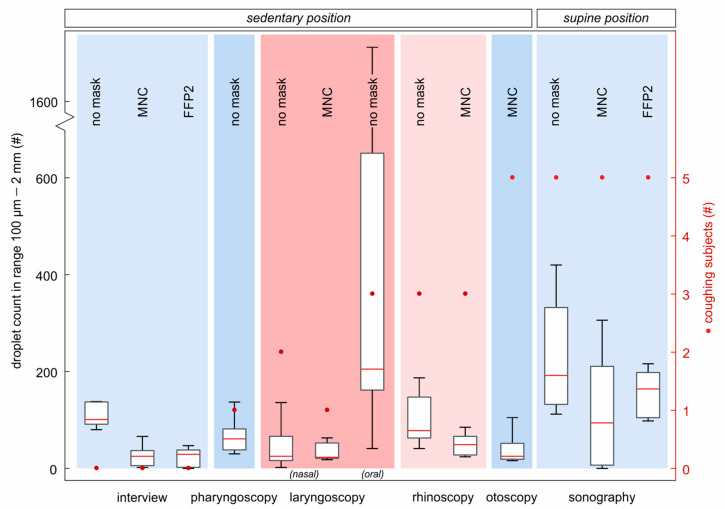
Boxplots of all 13 tasks, divided into sedentary und supine position tasks. The procedures are shown on the *x*-axis. The detectable droplet count, ranged between approx. 100 µm and 2 mm, is shown on the left *y*-axis and corresponds to the boxplots. The right *y*-axis shows the number of subjects coughing during the respective task.

The results of the high-speed measurements for detecting large droplets are shown in Figure 5. The *x*-axis is subdivided into the six ENT procedures. Tasks in sedentary position are shown on the left and in supine position on the right-hand side. The figure is sorted into tasks without coughing (patient interview), coughing induced by the procedure (pharyngoscopy/laryngoscopy/rhinoscopy) and intentional coughing by the subject (otoscopy/sonography). The left *y*-axis shows the number of detected droplets. The number of coughing subjects for every task is shown on the right *y*-axis.

Overall, the results show lower droplet emissions for tasks with MNC and FFP2 mask in comparison to the same tasks without mask. The median number of droplets detected during **patient interview** decreased when wearing an MNC by 73% and with an FFP2 mask by 71% (no mask: 101, MNC: 27, FFP2: 29).

**Pharyngoscopy** showed a median particle count of 61.

For **flexible nasal laryngoscopy** without mask, we detected a median of 25 droplets. However, when subjects were coughing during the procedure, values increased by over 440% (median: 25, coughing: 136). When they breathed calmly during the procedure, the detection rate was comparable to a masked task, e.g., patient interview. Flexible nasal laryngoscopy performed through an MNC showed a 9% decreased median value with lower variation, which is comparable to the interview with mask.

The highest particle generation was measured from rigid **oral laryngoscopy** with a median value 790% higher than the flexible nasal version and was accompanied by three coughing subjects. The maximum value thereby is a factor of 68 higher (1712 droplets). While performing the procedure, the physician held the subject’s tongue with a paper tissue which often stuck to the tongue mucosa. By removing the tissue, it often tore and created airborne particles smaller 0.5 mm that were also detected (Figure 3).

**Rhinoscopy** without mask resulted in median values approx. 60% higher than for the same task without mask (no mask: 78, FFP2: 49).

**Otoscopy** showed a median of 25 particles which is comparable to the other sedentary tasks with subjects wearing masks, e.g., patient interview, laryngoscopy and rhinoscopy.

**Sonography** was carried out in supine position. Compared to sedentary tasks, we measured overall higher droplet counts in the three supine tasks. Nevertheless, the relative decrease of detected particles for sonography with and without masks are comparable to those in sedentary position. During the tasks with MNC, the median number of particles decreased by 51% and thus is in a similar range as the interview which yielded a decay by 73%. With FFP2 mask, the median is 15% below sonography without mask.

### 3.2. Aerosol Particle Emission

**Figure 6:**
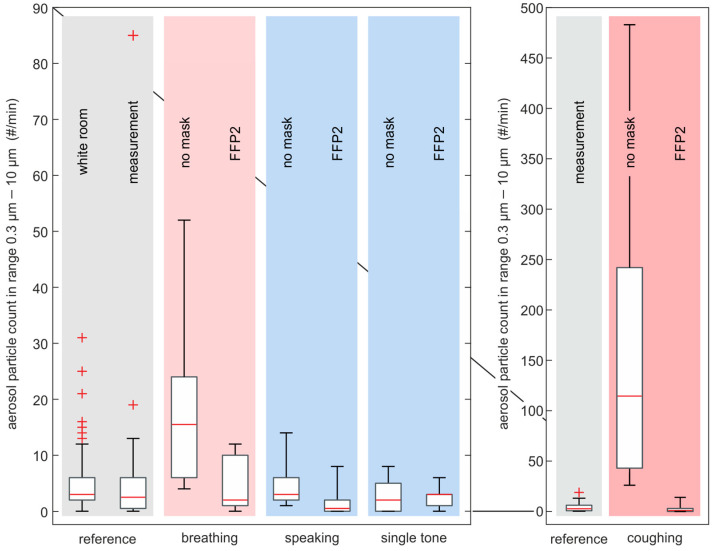
Boxplots of all aerosol particle measurement tasks performed in an OR. *Y*-axis shows the accumulated aerosol particle count in the range from 0.3 µm to 10 µm and *x*-axis shows the performed task. For better visualization, the diagram is divided into two plots with different *y*-axis limits. Note that the measurement references in both plots are the same. We use boxplots without outliers to show the data of the RAs, due to the small sample size of *n* = 6. The reference data sets are shown by boxplots with outliers (+), since they consist of larger data sets (540/24 measurements).

More than 87% of all the aerosols measured without a mask were found to be smaller than 1 µm. Table 3 shows the percentage size distribution of aerosol particles in the range from 0.3 µm to 10 µm divided into the four basic respiratory activities, averaged over all subjects. Figure 6 shows the absolute particle count in the range between 0.3 µm and 1 µm. The *x*-axis is subdivided into the four representative tasks, e.g., forced breathing, speaking, single tone vocalization (“/a/”), coughing and additionally the reference measurements. The coughing data is shown on a separate subplot with larger *y*-axis range to improve readability. The aerosol particle number is presented in counts per minute based on a passage flow of 1 liter/min through the OPS. In general, tasks with FFP2 mask showed a smaller median number of aerosol particles than tasks without a mask. The results for all tasks that involved wearing a mask were in the same order of magnitude as the background noise of the OR. This is also valid for speaking and single tone vocalization without a mask. For forced breathing without mask, we measured a median of 15.5 aerosol particles/min, which is approx. a factor of four higher than speaking and single tone vocalization. Highest values were measured for coughing without a mask with median values of 114.5 p/min and a factor of 7.4 higher than forced breathing.

## 4. Discussion

The results confirm that both MNC and FFP2 masks are an effective protection against aerosol particle and large particle emission during ENT procedures [30,31,32]. Both mask types reduced the particle emission down to the background noise level of the OR. This is valid for all performed tasks and measuring techniques. A difference between MNC and FFP2 masks was not observed in the **patient interview**. However, it cannot be excluded that droplets were occluded behind the subject and therefore not detected if they escaped through leakages at the cheeks. In contrast to aerosol particles, escapes of ballistic droplets through leakages is expected to be improbable as other study showed the high filtering effectiveness of ballistic droplets by surgical and FFP2/N95/KN95 mask types [15,33,34,35,36].

**Nasal flexible laryngoscopy** generated fewer large droplets compared to oral rigid laryngoscopy, both through a mask and without. Subjects also coughed less often during nasal flexible laryngoscopy than in **oral rigid laryngoscopy**, which means fewer particle generation. Aerosol and droplet emission are further reduced by performing the exam through a mask. Another advantage of nasal flexible laryngoscopy is that the physician has a greater distance to the patient and is therefore less exposed to the direct particle emission. Additionally, the patient can keep the mouth closed. Another critical issue in oral laryngoscopy is that many particles were dislodged from the paper tissue used to hold the patient’s tongue. Due to the previous contact with the patient’s tongue, these particles are most likely contaminated with virus particles and, thus, display a risk of virus transmission to the examiner. If oral rigid laryngoscopy is indicated, a non-dusting cloth such as gauze should be used to hold the patient’s tongue and prevent particles from becoming airborne. Since all three laryngoscopy techniques lead to the imaging of the vocal folds, the choice of technique should be part of the risk management for a physician’s self-protection. Our data suggests that without clinical necessities of oral rigid laryngoscopy, the nasal version is favorable as it can additionally be performed with a pierced MNC to further reduce the particle emission in case of coughing or sneezing.

**Pharyngoscopy** has increased particle output compared to mask tasks. Performing this procedure technique through a mask is not possible. The particles are expelled directly into the physician’s face. Hence, the physician should wear an FFP2 mask and a face shield to ensure maximal protection. Furthermore, he/she should reduce the time of the procedure as medically indicated as possible and try to avoid touching the tongue base in order to minimize choking or coughing, due to the close distance to the patients face.

**Rhinoscopy** without a mask generated more detectable droplets than the same task performed through a pierced MNC. Three of five subjects coughed during the exam with and without a mask. This and the lower droplet count indicate that it is safer for physicians to carry out this type of procedure through a pierced MNC which could be performed without further training.

**Sonography** was the only task in supine position and showed an overall offset in particle generation for the three tasks compared to the comparable sedentary procedures (interview/otoscopy) and is approx. a factor of five higher. We assume that the subject’s upper bodies reflect more light back into the region of interest (ROI) in supine position, which leads to higher detection rates for small particles. However, considering the offset, the ratio between the number of particles with mask to the number without mask is similar to the tasks in sedentary position.

**Otoscopy** presented as one of the least critical examinations regarding the expelled droplets in front of the patient. A recent study by Falk et al. (2021) simulated the aerosol particle diffusion around the head of coughing and breathing subjects wearing an MNC and found that particles escapes through leakages of the mask at the cheeks when not tightly attached to the face [15,37]. That is the area where the physicians face is located during an otoscopy. We suggest patients to wear FFP2 masks during this examination to minimize physician’s aerosol exposition, since surgical masks are known for leakages at the cheeks and the nose [15,36,38].

We found no studies tracking and counting large visible droplets during the performance of routine ENT procedures. Most groups concentrate on typical spontaneous RAs such as sneezing, coughing, speaking or breathing. On the other hand, ENT procedures often can be regarded as superposition of different RAs, although to a greater degree in form of forced breathing during laryngoscopy, gagging, sneezing or coughing, induced by the examiner’s intervention. In fact, our data revealed that coughing generates increased amounts of large droplets and aerosol particles compared to other RAs and low values when subjects wear masks. On the one hand, this is consistent with the results of many other studies that show an up to tenfold increase of ballistic droplets without mask [15]. On the other hand, by considering the aerosol particle counts, there are also some studies that confirm our results of a higher aerosol particle production rate for coughing [7,9], but also studies that reported similar amounts of aerosol particles compared to other RAs [26]. The reasons for these discrepancies are likely the high influence of environmental conditions on the results, different measuring setups and the highly individual production of aerosol particles depending on the emitter’s physiological health conditions as body hydration, hormonal state, age, BMI or infection/allergies in the respiratory tract [8,39].

Furthermore, the present data revealed a larger number of aerosol particles for breathing than for speaking and single tone phonation. This is contrary to most of other studies which reported mostly the smallest emission of aerosol particle for breathing [7,9]. A reason for this deviation from our study might be that the subjects were instructed to perform multiple short and forced exhalations as patients are instructed during laryngoscopy to avoid gagging. However, evaluating the ratio between counts for coughing compared to breathing, Morawska et al. reported a ratio of 7.4 measured in a clean room whereas the data presented here have different absolute particle counts but also yielded a ratio of 7 also measured in a clean room. This again shows high importance of controllable environmental conditions (temperature, particle-free, humidity) for counting respiratory aerosol particles.

Overall, the detection of respiratory particles (aerosol and ballistic droplets) is highly susceptible to environmental and measuring conditions, such as the choice of measuring device, visual conditions (illumination, contrast), temperature, ventilation, humidity and subject’s physiological state [40]. This makes a direct comparison of reported results for respiratory particles difficult. However, almost all studies found that coughing manifests predominantly as an RA with high or the highest emission of respiratory particles, both ballistic and aerosol ones. Indeed, this is very critical during ENT procedures that often provoke coughing combined with choking.

Summarizing, oral laryngoscopy led to the highest number of respiratory particles with the maximum number of particles emitting directly in the examiner’s face. Flexible nasal laryngoscopy should be favored as the examiner’s distance to the patients is larger and the number of respiratory droplets lower. Ideally, the flexible nasal endoscopy may be performed through a hole in a mask to ensure minimal aerosol production. Interestingly, speaking and coughing in a supine position (e.g., during sonography) without a mask also lead to a high number of respiratory droplets with a trajectory from bottom to top which is often underestimated in clinical practice. Even heavy breathing without mask through an examination yielded 22 aerosol particles per minute. These findings were measured in control subjects to ensure the safety of the research team. However, one can expect even a higher amount of respiratory particles, e.g., in patients with acute infections of the respiratory tract [41]. Simultaneously, this means that the results are independent of the current SARS-CoV-2 pandemic and that the results may be applied to any other airborne disease.

We analyzed the dispersion in the examination room of the presented experiments in a separate manuscript with numerical simulations [37]. The maximal distance of the aerosols travelled in the examination room was 3.4 meters (for particles < 2.241 µm) measured after 60 s. Thus, as aerosol particles remain in the air up to hours [42], airing the examination room after each patient encounter seems to be an easy and cheap way to reduce aerosol accumulation and ensure the safety of the next patients [43].

Our results and the results of the studies of other groups [26,44] suggest that ENT physicians should wear (e.g., FFP2 or equivalent) masks during the whole patient encounter, from taking history to the examination or in-office procedures in order to protect themselves independent of the pandemic. Eye protection/face shields may also be considered during close contact examinations that induce coughing. It even has to be critically considered if patients with respiratory infections or airborne diseases may be asked to wear masks during a physician encounter after the current pandemic in order reduce the aerosol burden in the room for the next patient. Maximal reduction of particle emission is key as even when patients were wearing FFP2 masks, particle emission was not zero.

### Limitations

The subject sample sizes of *n* = 5 for large droplets and *n* = 6 for aerosol particles presented in this study are not estimated. Therefore, the measurement results are presented descriptively, and only basic statistical measures are calculated. We use boxplots without outliers to present our task-based data. For references we use boxplots with outlier, since we performed more individual reference measurements, e.g., *n* = 540 for white room and *n* = 24 in between measurements. Additionally, the droplet measurements have not been conducted in a clean room environment, contrary to the aerosol measurements.

As mentioned in the discussion, the count for the sonography tasks with mask are approx. a factor of five higher than for the otoscopy task, which indicates a systematic difference in the measuring setup when analyzing different subjects’ positions. The reason might be a higher light intensity above the subject in the ROI leading to higher detection rates for smaller particles.

## 5. Conclusions

This study shows that in all examined ENT procedures both large particles in the mm range and aerosol particles in the sub µm range are expelled by the patient, which potentially carry a viral load and are dangerous for the physician. Rigid oral laryngoscopy produced the highest amount of aerosol particles right into the examiner’s face but also during sonography or otoscopy without a mask, aerosol particles were expelled close to the examiner and in the examination room. The exposure to aerosols and droplets and thereby the risk of infection of physicians can be reduced by deliberate choice of examination technique depending on medical indication and the use of appropriate equipment for the examiner (e.g., FFP2 masks) and the patients. As particle emission was not zero even when the patient wore an FFP2 mask, protective equipment for the examiner is key. As aerosol dispersion travels up to 3.4 m within 60 s, airing the room after each patient helps reducing the aerosol load for the next patient.

## Figures and Tables

**Table 1:** Overview of the large droplet experiments. The first column shows the analyzed ENT procedures, the second column which type of face masks were used by the subject during the procedure in sequential experiments and the third column shows the subject’s body positioning during the experiment.

ENT Procedure	Subject’s Face Mask	Subject’s Position
Patient interview	No mask|MNC|FFP2	Sedentary
Pharyngoscopy	No mask	Sedentary
Laryngoscopy (nasal)	No mask|MNC	Sedentary
Laryngoscopy (oral)	No mask	Sedentary
Rhinoscopy	No mask|MNC	Sedentary
Otoscopy	MNC	Sedentary
Sonography	No mask|MNC|FFP2	Supine

**Table 2:** Basic respiratory activities (RAs) analyzed with the aerosol particle test stand in the diameter range of 0.3–10 µm using an OPS. RAs were derived from the shown ENT procedures.

Equivalent Task	Subject’s Face Mask	Task Description	Derived From
Patient interview	No mask|FFP2	1 min of reading	Patient interview Sonography Otoscopy
Pharyngoscopy	No mask|FFP2	1 min with up to 25 forced exhalations	Pharyngoscopy Rhinoscopy Laryngoscopy
Laryngoscopy (nasal)	No mask|FFP2	1 min of coughing (approx. 25 coughs)	Otoscopy Laryngoscopy Rhinoscopy Pharyngoscopy
Laryngoscopy (oral)	No mask|FFP2	1 min at comfortable pitch and loudness of the single tone “/a/”	Laryngoscopy Patient interview

**Table 3:** Percentage size distribution of aerosol particles in the four basic respiratory activities in the diameter range from 0.3 µm to 10 µm. On average, more than 87% of the aerosol particles were smaller than 1 µm in the experiments without a mask and 74% with an FFP2 mask.

Aerosol Particle Diameter Range (µm)	Breathing (%)		Speaking (%)		Single Tone Vocalization (%)		Coughing (%)	
	No Mask	FFP2 Mask	No Mask	FFP2 Mask	No Mask	FFP2 Mask	No Mask	FFP2 Mask
0.3–0.37	15.1	10.3	20.7	3.8	17.6	21.1	29.2	20.9
0.37–0.47	23.5	10.3	24.1	11.5	23.5	15.8	23.1	18.6
0.47–0.58	21.8	10.3	20.7	7.7	11.8	0	16.6	16.3
0.58–0.72	6.7	13.8	6.9	34.6	17.6	5.3	8.2	9.3
0.72–0.9	8.4	10.3	3.4	7.7	0	10.5	6.5	11.6
0.90–1.12	9.2	3.4	3.4	7.7	5.9	5.3	5.8	7
1.12–1.39	3.4	0	0	3.8	0	15.8	3.2	4.7
1.39–1.73	0.8	3.4	0	0	0	5.3	1.5	2.3
1.73–2.16	2.5	6.9	3.4	3.8	5.9	0	2.8	2.3
2.16–2.69	2.5	6.9	0	7.7	11.8	0	1.3	0
2.69–3.34	1.7	6.9	0	0	0	5.3	0.7	0
3.34–4.16	0	3.4	0	0	0	0	0.5	0
4.16–5.18	0.8	3.4	0	3.8	5.9	0	0.2	0
5.18–6.45	1.7	3.4	0	3.8	0	5.3	0.1	4.7
6.45–8.03	0	3.4	0	0	0	5.3	0.3	0
8.03–10	0	0	0	0	0	0	0.1	0
<10	1.7	3.4	0	3.8	0	5.3	0	2.3

## Data Availability

Not applicable.
